# EBUS-TBNA in mediastinal staging of non-small cell lung cancer: comparison with pathological staging

**DOI:** 10.36416/1806-3756/e20230353

**Published:** 2024-07-29

**Authors:** Sara Braga, Rita Costa, Adriana Magalhães, Gabriela Fernandes

**Affiliations:** 1. Serviço de Pneumologia, Hospital Sousa Martins - Unidade Local de Saúde da Guarda E.P.E., Guarda, Portugal.; 2. Serviço de Cirurgia Torácica, Centro Hospitalar Universitário de São João E.P.E., Porto, Portugal.; 3. Serviço de Pneumologia, Centro Hospitalar Universitário de São João E.P.E., Porto, Portugal.

**Keywords:** Carcinoma, non-small cell lung, Neoplasm staging, Endoscopic ultrasound-guided fine needle aspiration

## Abstract

**Objective:**

Although EBUS-TBNA combined with EUS-FNA or EUS-B-FNA stands as the primary approach for mediastinal staging in lung cancer, guidelines recommend mediastinoscopy confirmation if a lymph node identified on chest CT or showing increased PET scan uptake yields negativity on these techniques. This study aimed to assess the staging precision of EBUS/EUS.

**Methods:**

We conducted a retrospective study comparing the clinical staging of non-small cell lung cancer patients undergoing EBUS/EUS with their post-surgery pathological staging. We analyzed the influence of histology, location, tumor size, and the time lapse between EBUS and surgery. Patients with N0/N1 staging on EBUS/EUS, undergoing surgery, and with at least one station approached in both procedures were selected. Post-surgery, patients were categorized into N0/N1 and N2 groups.

**Results:**

Among the included patients (n = 47), pathological upstaging to N2 occurred in 6 (12.8%). Of these, 4 (66.7%) had a single N2 station, and 2 (33.3%) had multiple N2 stations. The adenopathy most frequently associated with upstaging was station 7. None of the analyzed variables demonstrated a statistically significant difference in the occurrence of upstaging. PET scan indicated increased uptake in only one of these adenopathies, and only one was visualized on chest CT.

**Conclusions:**

Upstaging proved independent of the studied variables, and only 2 patients with negative EBUS/EUS would warrant referral for mediastinoscopy. Exploring other noninvasive methods with even greater sensitivity for detecting micrometastatic lymph node disease is crucial.

## INTRODUCTION

Lung cancer stands as the malignancy with the highest global mortality rate, and its incidence shows a continual rise.^1^ Accurate staging is pivotal for prognostic assessment and crafting suitable treatment plans, crucially determining whether the tumor is anatomically amenable to curative surgical resection.

In the absence of distant metastasis, curative treatment hinges on the extent of tumor involvement (T) and the level of thoracic lymph node engagement. Generally, for patients with N0 (no lymph node involvement) and N1 (metastasis in ipsilateral pulmonary or hilar lymph nodes) disease, along with a resectable primary tumor, surgery emerges as the preferred option, provided that the patient demonstrates satisfactory cardiopulmonary function and absence of high risk comorbidities. If contralateral mediastinal/hilar lymph nodes or supraclavicular nodes (N3) are involved, surgery is not typically pursued. However, in cases of ipsilateral mediastinal or subcarinal lymph nodes (N2) involvement, surgery may be considered as part of a multimodality treatment approach. The decision depends on factors such as the number, size, and location of the affected lymph nodes, determined through a multidisciplinary team consensus.^2^ ^,^ ^3^

EBUS-TBNA has evolved into an indispensable technique for lung cancer staging, emerging as the primary choice for approaching mediastinal lymph nodes.^4^ ^,^ ^5^ ^)^ The combined utilization of EBUS-TBNA and EUS-FNA or transesophageal bronchoscopic ultrasound-guided fine needle aspiration (EUS-B-FNA) is strongly recommended over relying solely on EBUS.^5^ EUS serves as a valuable complement to EBUS, synergistically enhancing the diagnostic yield of this staging approach.^5^ ^-^ ^7^ ^)^ Expanding beyond the stations accessed by EBUS alone (stations 2, 3, 4, 7, 10, 11), EUS extends accessibility to additional stations, including 2L, 4L, 5, 7, 8, and 9.^5^ ^-^ ^7^ This collaborative use of both techniques is progressively supplanting surgical approaches in lung cancer staging.^5^ ^-^ ^7^

For several years, mediastinoscopy held the prestigious status of the gold standard for mediastinal staging in lung cancer. However, contemporary guidelines relegate it to a minor role,^2^ a shift attributed not only to the rise of minimally invasive approaches but also to the limited access provided by mediastinoscopy to lymph node stations 2, 4, and 7.^8^ Beyond the conventional cervical mediastinoscopy, initially described in 1959,^9^ alternative techniques for invasive staging have emerged, including video-assisted mediastinoscopy (VAM), video-assisted mediastinoscopic lymphadenectomy (VAMLA), and transcervical extended mediastinal lymphadenectomy (TEMLA). VAMLA employs a mediastinoscope affixed to a camera and a blade, expanding the dissection space for systematic lymph node dissection, particularly targeting stations 2R, 2L, 4R, 4L, and 7 to enhance sensitivity.^10^ ^,^ ^11^ On the other hand, TEMLA represents the most extensive transcervical mediastinal dissection technique, incorporating sternal elevation and video mediastinoscopy. It provides access to a broader array of lymph node stations, including 1, 2R, 2L, 3a, 4R, 4L, 5, 6, 7, and 8. However, it is important to note that TEMLA carries a heightened risk of complications and mortality, a contrast to the comparatively lower risks associated with EBUS and EUS.^10^ ^,^ ^11^ However, as per the guidelines, if a mediastinal lymph node exhibits heightened uptake on PET or is detected on chest CT but yields a negative result on EBUS/EUS, the recommended course of action is surgical confirmation through mediastinoscopy.^2^ ^,^ ^12^

We conducted a retrospective analysis to assess the staging precision of EBUS/EUS. The primary objective was to compare the clinical staging of non-small cell lung cancer (NSCLC) patients who underwent EBUS-TBNA/EUS-B-FNA with their pathological post-surgical staging. The secondary objectives encompassed the evaluation of the impact of selected variables, such as histology, tumor location, size, and the time elapsed between EBUS and surgery on post-surgical staging. Additionally, the study aimed to compare the concordance between CT/PET results, EBUS/EUS findings, and the subsequent pathological staging after surgery.

## METHODS

This retrospective study was conducted at a tertiary hospital, spanning between January of 2019 and December of 2021.

### Population

Following the diagnosis and histological confirmation of NSCLC, all patients underwent a PET scan to evaluate the extent of the disease. In cases without distant metastases, patients with a primary tumor exceeding a large axis of > 3 cm, enlarged lymph nodes surpassing a short axis of > 1 cm on chest CT, adenopathies exhibiting increased uptake on PET scan, and those with centrally located primary tumors were chosen for lymph node staging through EBUS/EUS.

Patients with a mediastinal staging of N0 or N1 on EBUS/EUS, who underwent surgery, and had at least one biopsied station (with representative lymph node material) approached in both procedures were included in the study. Patients undergoing neoadjuvant therapy were excluded from the analysis.

Subsequently, the selected patients were categorized based on post-surgical pathological results into two groups: N0/N1 and N2. The 8th international staging system TNM was utilized for classification.

### EBUS-TBNA/EUS-B-FNA

EBUS-TBNA/EUS-B-FNA procedures were conducted under either intravenous general anesthesia or intravenous sedation, coupled with local anesthesia using 2% lidocaine.

Both techniques were performed with a flexible ultrasound bronchoscope (BF-UC180F; Olympus, Japan or BF-UC190F; Olympus, Japan) equipped with a convex ultrasound transducer (7.5 MHz). Image manipulation was facilitated through a dedicated ultrasound processor (EVIS EXERA III [CLV-190]; Olympus, Japan) and color power Doppler mode was used when necessary. The punctures during the procedure were executed using a 22-gauge needle (NA-201SX-4022; Olympus, Japan). All examinations were carried out by bronchoscopists well-versed in this procedure, boasting more than five years of experience.

Upon introducing the bronchoscope through a mouthpiece in the tracheobronchial tree, lymph node mapping was initiated. Following the identification of adenopathies, sample collection commenced, starting from the contralateral nodes and progressing from N3 to N1.

EUS-B was employed for CT/PET positive adenopathies that proved challenging or inaccessible via EBUS. During EUS-B, the bronchoscope was introduced into the esophagus, identifying and evaluating lymph node stations from the lowest (9) to the highest (2L), as previously described.^13^ In cases in which more than one adenopathy was punctured, the procedure extended from station N3 to N1. A minimum of three needle passes were executed for each station.

Rapid on-site evaluation was not conducted. The aspirated samples were deposited in a container with a preservation solution, facilitating the subsequent preparation of a cytoblock for further cytological examination.

### Surgery

Surgeries were conducted utilizing the uniportal VATS technique, following the established standard procedure as previously described.^14^ This technique was performed under general anesthesia, employing selective ventilation with a double-lumen tube. The radical lymphadenectomy encompassed the N1 hilar nodes of the respective lung (stations 10 and 11). For N2 stations, radical lymphadenectomy targeted stations 7, 8, and 9 on both sides; stations 2, 3, and 4 on the right side; and stations 5 and 6 on the left side.

### Statistical analysis

Data presentation included absolute and relative frequencies for categorical variables, while mean and standard deviation or median and range were applied for continuous variables.

The statistical analysis employed included the chi-square test and the Fisher’s exact test for independent samples, results being considered statistically significant if p < 0.05. Statistical analyses were carried out using IBM SPSS Statistics software package, version 26 (IBM Corporation, Armonk, NY, USA).

## RESULTS

**Figure 1:**
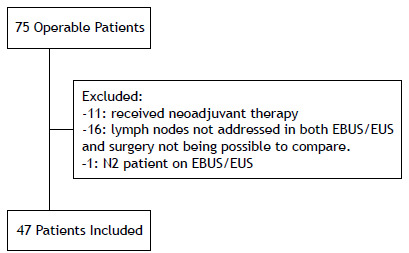
Selection of the patients included in the study.

Our initial sample consisted of 75 patients who underwent surgery following endosonographic mediastinal staging. Of these, 47 were considered for inclusion in the study, while 28 were excluded. Exclusion criteria comprised the administration of neoadjuvant therapy (n = 11), cases in which lymph nodes were biopsied on EBUS/EUS without subsequent surgical approach (n = 16), and presence of N2 stage on EBUS/EUS (n = 1; Figure 1).

Among the 47 patients included, 41 (87.2%) were male, with ages ranging from 47 to 85 years (median = 69 years; Table 1). Concerning tumor histology, 34 (72.3%) and 13 (27.7%) were diagnosed with adenocarcinoma and squamous cell carcinoma, respectively. Regarding tumor location, 29 tumors (61.7%) were situated in the right lung, most of which (n = 29; 61.7%) being located in the upper lobes. Among these patients, 6 (12.8%) had tumors in a central location (within the inner two-thirds of the lung), and 41 (87.2%) had them in peripheral locations. Tumor size varied from 9 to 66 mm (median = 29 mm).

**Table 1:** Descriptive analysis of the variables under study.

Variables	n (%)
Gender	
Male	41 (87.2)
Female	6 (12.8)
Histology	
Adenocarcinoma	34 (72.3)
Squamous cell carcinoma	13 (27.7)
Pulmonary side of the tumor	
Right	29 (61.7)
Left	18 (38.3)
Pulmonary lobe of the tumor	
Upper lobe	29 (61.7)
Lower lobe	16 (34.0)
Upper and lower lobes	1 (2.1)
Upper and middle lobes	1 (2.1)
Location of the tumor	
Central	6 (12.8)
Peripheral	41 (87.2)

Age, years: median = 69 [range, 47-85]; mean = 67.66 ± 9.30.

Tumor size, mm: **median** = 29 [range, 9-66]; mean = 29.81 ± 13.30.

As for noninvasive staging methods, regarding the presence of adenopathies on CT (with a size greater than 10 mm in the short axis), 31 patients (66%) were staged as N0/N1, while 16 (34%) were classified as N2/N3. Regarding the presence of adenopathies with increased uptake on PET scan, 22 (46.8%) of the patients were staged as N0/N1, and 25 (53.2%) of the patients were categorized as N2/N3.

In the context of EBUS-TBNA and EUS-FNA, a total of 378 punctures were performed, with a mean of 9.0 ± 3.2 punctures per patient (ranging from a minimum of 3 to a maximum of 16). The median number of punctures per lymph node station was 3. The mean size of the biopsied lymph node was 8.5 mm, spanning from 4.5 mm to 15 mm. The most frequently biopsied lymph node was station 7 (n = 39), followed by station 4R (n = 31) and 4L (n = 20). There were no complications during the EBUS/EUS performance.

Concerning surgery outcomes, the time interval between EBUS and surgery varied from 30 days to 280 days (median = 68). The patient with the lengthiest gap between the two procedures underwent transthoracic needle biopsy (TNB), which did not yield a diagnosis. Subsequently, EBUS/EUS was performed while awaiting the TNB result, and it turned out to be negative. Only later was the patient able to undergo a repeat TNB, confirming the diagnosis of neoplasia, followed by surgery. There was one intraoperative complication involving major bleeding that necessitated conversion to thoracotomy. No other major complications were observed.

Among the patients included, pathological staging revealed N0/N1 in 41 patients and N2 in 6 patients. Consequently, upstaging to N2 occurred in 6 (12.8%) of these patients. Of the upstaged patients, 4 (66.7%) had only one N2 station, while 2 (33.3%) had multiple N2 stations (in both cases, 4R and 7). The adenopathy most frequently associated with upstaging was station 7 (in 4 patients), followed by adenopathy 4R (in 3 patients; Table 2). The average size of tumors in upstaged patients was 28.2 mm.

**Table 2:** Characteristics of the upstaged patients.

Case	Station	Histology	Size (mm)	Location	Side	Lobe	PET positivity	CT positivity
1	7	Adenocarcinoma	38	Peripheral	Left	Lower	No	No
2	5	Adenocarcinoma	13	Peripheral	Left	Upper	No	No
3	4R	Adenocarcinoma	15	Peripheral	Right	Upper	Yes	No
4	4R/7	Adenocarcinoma	25	Peripheral	Right	Upper	No/No	No/No
5	7	Adenocarcinoma	29	Peripheral	Left	Lower	No	No
6	4R/7	Adenocarcinoma	49	Peripheral	Right	Upper	No	Yes/No

All upstaged patients had adenocarcinoma, with no statistically significant difference regarding this variable. As for the lung side and lobe involved, there was no statistically significant difference regarding upstaging. Upstaging occurred in 3 (10.3%) of the patients whose tumor was located on the right side and in 3 (16.7%) of the patients whose tumor was located on the left side. Regarding the lobe involved, upstaging occurred in 4 (13.8%) of the patients whose tumor was located in the upper lobe, in 2 (12.5%) patients whose tumor was located in the lower lobe, and in none of the patients with involvement of more than one lobe (Table 3). Of the patients with a peripheral tumor location, 6 (14.6%) had upstaging, while among those with a central tumor location (n = 6), none had upstaging, with no statistically significant difference regarding this variable. Regarding both the time between EBUS and surgery and tumor size, there was no statistically significant difference regarding upstaging.

**Table 3:** Association between the variables studied and upstaging.

Variable		Upstaging		p
		Yes	No	
Histology	Adenocarcinoma	6	28	0.1
	Squamous cell carcinoma	0	13	
Pulmonary side where the tumor is located	Right	3	26	0.7
	Left	3	15	
Pulmonary lobe where the tumor is located	Upper	4	25	0.96
	Lower	2	14	
	Upper and lower	0	1	
	Upper and middle	0	1	
Location of the tumor	Central	0	6	0.4
	Peripheral	6	35	
Time between EBUS and surgery, months	< 3	3	29	0.37
	≥ 3	3	12	
Tumor size, cm	< 3	5	19	0.1
	≥ 3	1	22	

Among upstaged patients, only 1 (16.7%) had increased uptake of the respective station on PET scan, and only 1 (16.7%) had the respective adenopathy visualized on chest CT.

Concerning staging based on imaging methods, in comparison with post-surgical staging, CT upstaging occurred in 5 (10.6%) of the patients, and downstaging occurred in 15 (31.9%) of the patients. As for PET scan, upstaging occurred in 4 (8.5%) of the patients, and downstaging occurred in 23 (48.9%) of the patients.

## DISCUSSION

Among the 47 patients with NSCLC included in the study, upstaging to N2 occurred in 6 (12.8%). The rates of upstaging to N2 can vary across different studies. For instance, Zirafa et al.^15^ conducted a comparison between lobectomy performed via open surgery and robotic surgery, reporting upstaging to N2 in 9.4% of patients with robotic lobectomy and 2.8% of patients with open lobectomy. Nachira et al.^16^ observed upstaging to N2 in 4.3% of cases with open surgery and in 13% using uniportal VATS. In that study,^16^ as in ours, no independent risk factor for upstaging was identified.

The decision to perform surgical resection in cases of known stage IIIA/B-N2 disease remains a subject of debate due to the heterogeneous nature of this group. Consequently, all such patients are presented at a multidisciplinary team meeting, where a thorough risk/benefit discussion takes place. The selection of treatment depends not only on imaging factors, such as the location, size, and number of lymph node stations involved,^12^ ^,^ ^17^ ^,^ ^18^ but also on the patient’s comorbidities and lung function.^19^ A retrospective study conducted by Mainguene et al.^17^ revealed that the histological type also plays a role in influencing treatment decisions. Adenocarcinoma, for instance, tends to be more commonly associated with surgical treatment, unlike squamous cell carcinoma, which is often linked to heavy smokers exhibiting greater functional limitations, a more central presentation, and more extensive lymph node involvement. In addition to these considerations, patient preference also plays a significant role in the treatment selection process.^18^ ^)^ The heterogeneity of this stage, coupled with the absence of a universal definition of resectability, complicates decision-making in multidisciplinary meetings. Mainguene et al.^17^, who examined the reproducibility of multidisciplinary decisions in IIIA/B-N2 disease, a 70% agreement was observed, with a kappa coefficient of 0.43. The 9th international TNM staging system classifies N2 stages into N2a (single station) and N2b (multiple stations), emphasizing the distinct prognosis associated with these categories. While this classification may contribute to informed treatment decisions, it also necessitates enhanced precision from radiologists, pathologists, and practitioners involved in performing mediastinal staging techniques.^20^

In potentially resectable stage IIIA/B-N2 cases (excluding T4), patients undergo neoadjuvant chemotherapy with or without radiotherapy. Following reassessment and absence of disease progression, these patients may be considered for surgical resection. For cases deemed nonresectable, the proposed course of action involves definitive chemoradiotherapy.^2^ ^,^ ^12^ ^)^ Regarding the 6 patients with post-surgical upstaging, 4 with only one N2 station had these lymph node metastases been detected before surgery, and their cases would likely have been discussed in a multidisciplinary team meeting, potentially opening the possibility of surgical resection.

Concerning PET scan staging, upstaging was observed in 4 patients (8.5%), while downstaging occurred in 23 (48.9%) of the patients. Recent studies have highlighted the high sensitivity of this test in detecting neoplastic disease but have also emphasized its low specificity.^21^ Interestingly, PET scan demonstrated increased uptake in only one of the adenopathies associated with pathological upstaging, and only one of these adenopathies was visualized on chest CT. In essence, most cases in which upstaging occurred were likely instances of micrometastatic disease, involving small clusters of tumor cells that went undetected in both imaging and endoscopic examinations.^22^ ^)^ Consequently, these cases may not have been suitable for referral to mediastinoscopy either.

The approach by EBUS-TBNA and EUS-B-FNA represents a minimally invasive and safe method, offering access to a greater number of lymph node stations when compared with mediastinoscopy. Yasufuku et al.^23^ demonstrated a combined sensitivity and negative predictive value of EBUS-TBNA and mediastinoscopy at 92% and 96%, respectively, supporting the notion that mediastinoscopy may not provide significant advantages after a negative EBUS. Liberman et al.^24^ concluded that the utilization of a combined EBUS/EUS procedure surpasses mediastinoscopy in the preoperative staging of NSCLC and should be considered the new gold standard in mediastinal staging, eliminating the need for confirmation by surgical staging in the case of negative EBUS/EUS results. A systematic review and meta-analysis^25^ have yielded the conclusion that both EBUS-TBNA and mediastinoscopy demonstrate comparable results in the mediastinal staging of NSCLC. Additionally, the study suggests that EBUS-TBNA can serve as a viable replacement for mediastinoscopy in patients with potentially resectable NSCLC. The authors assert that the complication rate associated with mediastinoscopy appears to be higher than that of EBUS-TBNA.^25^ In a study conducted by Ge et al.,^26^ a comparison between VAM and EBUS-TBNA for mediastinal staging resulted in the finding that both procedures similarly exhibit high diagnostic accuracy. However, the VAM group displayed a higher incidence of complications and fewer false negatives compared with the EBUS-TBNA group. Decaluwé et al.^27^ conducted a study where VAMLA emerged as the preferred technique for pre-resection mediastinal nodal staging in patients with cN1 NSCLC, as opposed to endosonography, owing to its high sensitivity. While VAMLA and TEMLA demonstrate high accuracy and reduced false negatives concerning potential micrometastases, the available data on their results and safety remain limited.

Recently, molecular biology techniques such as PCR and immunohistochemistry have been explored and applied in the detection of lymph node micrometastasis in NSCLC.^22^ The emergence of these new techniques holds significant importance for accurate staging, and, consequently, treatment strategies, as well as enabling a better prognostic assessment of patients with NSCLC. This detection could lead, for instance, in some cases, to neoadjuvant/adjuvant therapy, and, in others, to a multidisciplinary decision on nonsurgical treatment, thus not subjecting patients to unnecessary treatment, although more research is needed in this area.

This study has several limitations. Being retrospective, it restricts access to and analysis of certain variables and clinical characteristics. Furthermore, the small sample size could introduce potential bias into the study results and may impact the interpretation of statistical significance of certain variables. Additionally, there might be a selection bias, as patients who underwent neoadjuvant treatment were excluded, and those without at least one adenopathy approached in both procedures were also excluded. This exclusion criterion might have contributed to an underestimation of the upstaging value, although they align with findings from other studies. To address these limitations, future research efforts should consider conducting a prospective, multicenter study with a larger and more diverse sample of patients. That study should include participants who underwent neoadjuvant treatment, providing a more comprehensive understanding of the complexities associated with the staging of NSCLC. Furthermore, our study did not make a direct comparison with mediastinoscopy, which would also be interesting in a future study in which other techniques such as VAMLA, TEMLA, and their respective accuracy and complications could be evaluated.

In conclusion, our study showed N2 upstaging in 12.8% of the sample, a result consistent with other studies. The upstaging in this study was determined to be independent of the variables under investigation. Only two patients, both with negative lymph nodes on EBUS/EUS that showed positivity on either PET or CT would potentially be candidates for referral to mediastinoscopy.

This study not only presents findings corroborated by previous studies but also delves into contentious aspects surrounding these findings. We address the challenges associated with staging and treatment selection in stages IIIA/B-N2 and the inherent variability in multidisciplinary decision-making processes, exploring the potential benefits offered by the latest 9th international TNM staging system in effectively categorizing stage N2. Moreover, the article delves into the significance of PET in mediastinal staging and emphasizes the criticality of detecting micrometastatic disease. It also discusses the pivotal role played by advanced techniques such as EBUS/EUS, VAMLA, and TEMLA, and the importance of exploring other noninvasive methods with even greater sensitivity in achieving this goal.
